# Atomic-level insight into mRNA processing bodies by combining solid and solution-state NMR spectroscopy

**DOI:** 10.1038/s41467-019-12402-3

**Published:** 2019-10-04

**Authors:** Reinier Damman, Stefan Schütz, Yanzhang Luo, Markus Weingarth, Remco Sprangers, Marc Baldus

**Affiliations:** 1 NMR Spectroscopy, Bijvoet Center for Biomolecular Research, Utrecht University, Padualaan 8, 3584 CH Utrecht, The Netherlands; 2 Department of Biophysics I, University of Regensburg, 93053 Regensburg, Germany

**Keywords:** Protein aggregation, RNA, Intrinsically disordered proteins, NMR spectroscopy

## Abstract

Liquid–liquid phase separation is increasingly recognized as a process involved in cellular organization. Thus far, a detailed structural characterization of this intrinsically heterogeneous process has been challenging. Here we combine solid- and solution-state NMR spectroscopy to obtain atomic-level insights into the assembly and maturation of cytoplasmic processing bodies that contain mRNA as well as enzymes involved in mRNA degradation. In detail, we have studied the enhancer of decapping 3 (Edc3) protein that is a central hub for processing body formation in yeast. Our results reveal that Edc3 domains exhibit diverse levels of structural organization and dynamics after liquid–liquid phase separation. In addition, we find that interactions between the different Edc3 domains and between Edc3 and RNA in solution are largely preserved in the condensed protein state, allowing processing bodies to rapidly form and dissociate upon small alterations in the cellular environment.

## Abstract (web summary)

Processing bodies are membrane less organelles that contain enzymes involved in mRNA turnover, among them enhancer of decapping 3 (Edc3). Here the authors use solid- and solution-state NMR spectroscopy to characterize the structural organization and dynamics of Edc3 and find that its interactions with RNA and between the different Edc3 domains are largely preserved in the phase-separated state.

## Introduction

Increasing evidence shows that cells develop intracellular protein-rich organelles that grow and fuse, allowing certain molecules to become locally enriched while excluding others^1^. Intracellular liquid–liquid phase separation (LLPS) processes seem to be a common route to achieving spatial organization of cellular components into dynamic, membrane-less compartments. While physical models have helped to understand the formation of membrane-less protein compartments^2^, studying such networks at atomic resolution has been challenging owing to the their intrinsic mobility and heterogeneous nature^3,4^ and because genetic approaches in a cellular context are complicated by the highly redundant nature of the clustering process.

NMR spectroscopy has been shown to provide unique structural insights into heterogeneous and dynamical systems at atomic resolution^5–8^. Previously, NMR in pure solutions has been used to decipher molecular interactions among LLPS components under in vitro conditions using titration studies^9,10^ and determined 3D structures of fast tumbling folded subdomains^11^. However, studying the structural organization of liquid-like droplets using solution-state NMR methods is complicated by the reduced diffusion^12^ of the LLPS molecules.

Hence, direct insights into the structural and dynamical organization of liquid-like droplets has been limited^12^. Instead, solid-state NMR (ssNMR) has been employed to study formation^13,14^ and structural organization^13,15–19^ of protein hydrogels and fibrillar assemblies that may be closely related to LLPS^3,20^. For example, we have previously used ssNMR to investigate the hydrogel state of the FG repeat domain of the nucleoporin Nsp1p, revealing that transient amyloid-like β-sheet interactions among NTQS-rich protein regions are responsible for gelation and network formation^13,16^. Such on-pathway amyloid interactions were also proposed for hydrogel-forming peptides that contain GSY-amino-acid-rich stretches and, in isolation, form crystalline needles reminiscent of amyloid fibrils^21^. Indeed, ssNMR revealed characteristic β-strand arrangements in fibrils formed by the low complexity domain of the FUS RNA-binding protein^18^. A relationship between LLPS and fibril-like protein states and their relevance for human disease has also been examined in stress granules composed of RNA-binding proteins and RNA^22^ and, more recently, for huntingtin exon1 assemblies in mammalian cells and yeast^23^. Although LLPS and fibrillization may represent two mechanistically distinct processes, models that connect these two processes have been developed^20,22,24^. For example, Molliex et al.^22^ have depicted the relationship between phase separation, fibrillization, and pathological inclusions by three steps with an intermediate maturation regime where two phases co-exist in the granule state, which ultimately connects LLPS to disease. However, the structural relationship between LLPS and maturation has remained elusive.

**Fig. 1:**
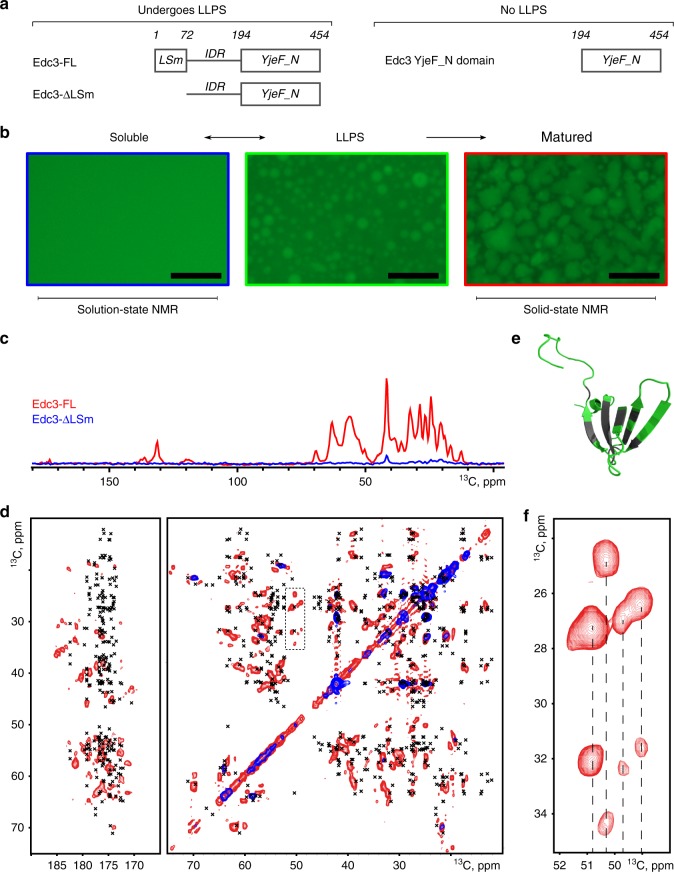
The LSm domain of Edc3 remains mobile after LLPS. **a** Overview of the Edc3 constructs used in this study. Residue numbers are indicated on top. **b** Fluorescence microscopy images of the three states of Edc3 that we study here. The left panel (blue box) indicates the soluble form of the protein (or protein:RNA complex). This state is assessed using solution-state NMR spectroscopic methods. Under specific conditions (see below), the soluble Edc3 protein can undergo LLPS to form liquid-like foci (middle panel, green box). These foci mature over time to form a gel-like state that no longer dissolves upon dilution or by increasing the salt concentration (right panel, red box). The matured state of Edc3 is studied using solid-state NMR methods. The length of the scale bar in the images is 50 μm. **c** HC INEPT spectra highlighting dynamic regions in the full-length phase-separated Edc3 protein (Edc3-FL, red) and in the phase-separated Edc3 protein that lacks the N-terminal LSm domain (Edc3-∆LSm, blue). **d** Two-dimensional C-C TOBSY spectra of the phase-separated Edc3-FL (red) and Edc3-∆LSm (blue) proteins. The crosses indicate the solution chemical shifts of the LSm domain. **e** The NMR structure of the LSm domain color-coded for residues where the solution-state chemical shifts match with resonances in the C-C TOBSY spectrum (green) and for residues where the solution-state chemical shifts are not in a region where there are resonances observed in the C-C TOBSY spectrum (gray). **f** Zoom on the proline region highlighted by the black dashed box in **d**, indicating four unique proline spin systems

In the following, we combined solid- and solution-state NMR experiments to directly study the LLPS and maturation of processing bodies (P-bodies) that are dynamic cytoplasmic ribonucleoprotein (RNP) granules containing proteins that are involved in translational repression and mRNA degradation^25–28^. Their main constituents are mRNA and the mRNA decay machinery, including the Dcp1:Dcp2 mRNA decapping complex, the RNA helicase Dhh1, the Pat–Lsm1–7 complex, the exonuclease XrnI and the Edc3^25,29^. Edc3 has emerged as a central hub for P-body formation in yeast. The 50 kDa protein (Fig. 1a) comprises an N-terminal LSm domain that directly interacts with helical leucine-rich motifs (HLMs) in the P-body proteins Dcp2^30^ and Pdc1^9^. C-terminal to the LSm domain, Edc3 contains a 120 amino-acid long intrinsically disordered region (IDR). This IDR is an interaction hub for the RNA helicase Dhh1^31^, RNA, and the C-terminal Edc3 YjeF_N dimerization domain^25^.

Previously, we showed that it is possible to reconstitute P-body-like assemblies in vitro using purified components^9,25^. These Edc3-containing in vitro foci undergo a maturation process, which over time results in the irreversible formation of an insoluble gel-like state (Fig. 1b). Based on solution-state NMR and phase separation diagrams, we identified key interactions in this sequential two-step process and found that interactions between the Edc3 LSm domain and HLMs in Dcp2 and interactions between the Edc3 IDR and RNA are required for LLPS. Recently, we noticed that the isolated Edc3 protein is also able to undergo LLPS and we have shown that the IDR and the YjeF_N domain are key for this process. Moreover, the ability of Edc3 to mediate phase transitions is significantly enhanced by RNA, which can bridge between Edc3 protomers through interactions with the Edc3 IDR^25^.

Solution-state NMR spectroscopy is ideally suited to study proteins and protein:RNA complexes before they undergo LLPS (Fig. 1b; left panel), however, it is less optimal to study full-length P-body components after phase separation (Fig. 1b, middle panel), owing to the limited diffusion within the high-density phase and owing to the fact that these in vitro P-bodies mature into a more solid-like phase that does not spontaneously dissolve into a liquid phase upon dilution^25^ (Fig. 1b, right panel). To study these states, we here turned to ssNMR.

In the following, we conducted ^13^C/^15^N- as well as ^1^H-detected ssNMR experiments using a combination of scalar- and dipolar-based correlation methods that, as we have shown previously^13,16,32^, provide a powerful tool to separate ssNMR protein signals of mobile and rigid protein regions. For ^1^H-detected ssNMR experiments, we furthermore made use of proton dilution approaches^33,34^ to increase spectral resolution under high-field/high-speed magic angle spinning (MAS) conditions. We applied our strategy to the matured state of the full-length Edc3 protein as well as to the matured state of an Edc3 construct that lacks the N-terminal LSm domain (ΔLSm, Fig. 1a, b) representing the minimal Edc3 construct that can undergo LLPS^25^. In addition, we examined the C-terminal YjeF_N domain by both solid- and solution-state NMR and conducted ssNMR experiments on Edc3 preparations after the addition of RNA, which significantly enhances the LLPS process of Edc3.

Our studies reveal that the intra- and intermolecular contacts that are present between the Edc3 protein and RNA before LLPS are also existing in the matured granule state of in vitro P-bodies. These interactions are diverse and range from weak electrostatic to hydrophobic contacts and include folded protein domains as well as IDRs.

## Results

### The LSm domain of Edc3 is mobile in the matured state

We initially examined the global dynamics of the matured state of full-length Edc3 (Edc3-FL; Fig. 1a, b) using scalar-based ssNMR experiments^13,16,32^. To this end, we recorded HC INEPT^35^ (Fig. 1c) and C-C TOBSY^36^ (Fig. 1d) spectra that reveal regions of the protein that are highly mobile. These spectra indicate that at least one subdomain of the full-length Edc3 protein remains mobile, even in the matured (gel-like) state. As we previously showed that the Edc3 LSm domain is not essential for the LLPS process of isolated Edc3^25^ (vide infra), we speculated that this domain might remain flexible in the matured P-body state. To test this hypothesis, we repeated our scalar-based ssNMR experiments on matured P-bodies that were prepared from the Edc3 protein that lacks the LSm domain (Edc3-∆LSm). In these spectra (Fig. 1c, d; blue) we observed a near-complete reduction of the dynamics that were visible for the Edc3-FL protein. In particular, backbone correlations were strongly reduced in two-dimensional C-C TOBSY spectra of the matured states of the Edc3-∆LSm proteins compared with Edc3-FL (Fig. 1d). Many of these disappearing correlations in Fig. 1d matched with previous solution-state NMR assignments of the isolated LSm domain and the corresponding residues are indicated in Fig. 1e in green. Deletion of the LSm domain removes two proline residues from the amino-acid sequence. Yet, for Edc3-∆LSm, a total of four proline resonances disappeared (Fig. 1f), suggesting that the presence of the highly mobile LSm domain increases the flexibility of a part of the IDR. From these data, we conclude that the Edc3 LSm domain remains highly flexible in the matured state of the Edc3 protein. This notion also implies that the LSm domain is accessible for interactions with other P-body proteins and that it can thus engage in interactions with Dcp2, as we previously showed^30^.

### The YjeF_N domain forms a rigid core in the matured state

**Fig. 2:**
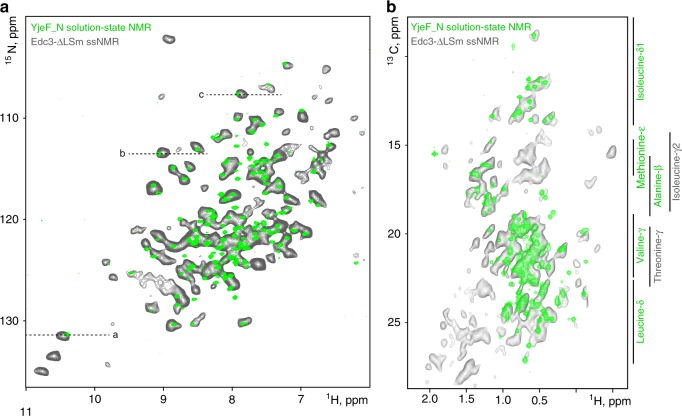
The fold of the YjeF_N dimerization domain is conserved after LLPS. Comparison of dipolar NH ssNMR spectra recorded on Edc3-∆LSm (gray) and of (^1^H-^15^N or methyl ^1^H-^13^C) TROSY-based spectra recorded with solution-state NMR on the YjeF_N domain (green). **a** ^1^H-^15^N region of the spectrum and **b** methyl region of the spectrum. The overall similarity between the spectra shows that the Edc3 Yjef_N domain is folded the same in solution and in the matured state. As a reference, average ^13^C chemical shifts for methyl-containing residues are indicated next to ^1^H-^13^C spectrum (note that Thr-γ, Ile-γ2 methyl groups and CH2 groups are not NMR-active in the solution-state samples). Dashed lines indicate the resonances used for 1D slices represented in Supplementary Fig. 2

The C-terminal YjeF_N domain of Edc3 forms a strong dimer interface^37^ that does not dissociate over time (Supplementary Fig. 1) and that is essential for the LLPS behavior of Edc3^25^. Solution-state NMR ^1^H-^15^N TROSY spectra of the isolated and soluble YjeF_N domain display a well-folded protein (Fig. 2a, green). To assess if the fold of the YjeF_N domain changes upon maturation of the phase-separated Edc3 protein, we recorded ^1^H-detected dipolar NH ssNMR spectra on the matured state of the Edc3-∆LSm protein (Fig. 2a, gray). These spectra display Edc3 regions that are rigid on the time scale of our ssNMR experiment. As the Yjef_N domain comprised the majority of the Edc3-∆LSm protein, we thus expected to observe mainly resonances from the dimerization domain. Indeed, we found a remarkable overall agreement between the NH correlation spectrum of the isolated Yjef_N domain in solution and the matured form of the Edc3-∆LSm construct (Fig. 2a. Supplementary Fig. 2, green v/s gray). The same agreement was observed for ^1^H-^13^C methyl TROSY^38^ spectra recorded on the soluble YjeF_N domain as compared with a ^1^H-detected dipolar CH ssNMR spectra recorded on the matured state of Edc3-∆LSm (Fig. 2b, green v/s gray). Note that the solution-state NMR spectrum in Fig. 2b only comprises signals of methyl groups from alanine-β, isoleucine-δ_1_, methionine-ε, valine-γ, and leucine-δ positions, as the other atoms are NMR inactive (see Methods).

Even in the absence of residue-specific assignments, this strong resemblance of solution- and ssNMR data suggests that the fold of the YjeF_N domain is largely conserved in the matured state of the Edc3 protein without major structural rearrangements. This notion rules out a scenario where the Yjef_N domain forms β-sheet-rich fibril-like structures after phase separation and maturation (Fig. 1b) and is further supported by the strong correlation between ssNMR spectra of the matured phase-separated Edc3-∆LSm protein and the lyophilized Yjef_N domain (Supplementary Fig. 3).

**Fig. 3:**
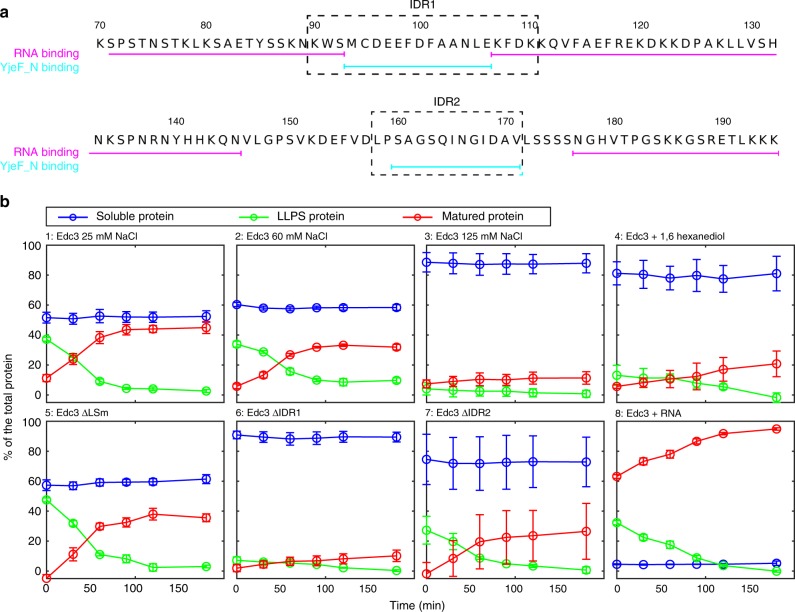
Edc3 LLPS and maturation. **a** Edc3 IDR sequence with highlighted residues that interact with the YjeF_N domain (cyan lines) and RNA (magenta lines). **b** Under different conditions, the fraction of total Edc3 protein that is in the soluble phase (blue lines; Fig. 1b, left panel), the LLPS phase (green lines; Fig. 1b, middle panel) and in the matured phase (red lines; Fig. 1b, right panel) were determined right after preparation of the solution and after 30, 60, 90, 120, and 180 minutes. Note that the decrease in the fraction of the protein that is in the LLPS phase correlates very well with the increase in the fraction of the protein that resides in the matured phase. This underscores that Edc3 maturation is a direct consequence of Edc3 LLPS (see Fig. 1b). Mean values and error bars (s.d.) are derived from three independent measurements. Protein concentrations are provided as a Source Data file

Despite the strong correlation between the ssNMR spectrum of the Edc3 protein in the phase-separated state and the solution-state NMR spectrum of the isolated Yjef_N domain, there are some notable differences. The major cause of this lies in the fact that the ssNMR spectrum was recorded on a protein construct that contains the IDR and the Yjef_N regions, whereas the solution-state NMR spectrum was obtained on a sample that only contains the Yjef_N domain. Note that it is not possible to record solution-state NMR spectra on a Edc3 construct that includes the IDR, as both Edc3-FL and Edc3-ΔLSm undergo LLPS and will transition into a matured state over time (see below), which is incompatible with solution-state NMR methods (Fig. 1b). Owing to the presence of the IDR in the sample that was used to record the ssNMR spectra, additional NMR resonances are expected and observed (Fig. 2a, gray v/s green spectrum). In addition, the IDR is known to interact with the Yjef_N domain in the phase-separated protein (Fig. 3a and see below). This will result in chemical shift perturbations of a set of the Yjef_N resonances and thus in differences between the solution and ssNMR spectra. In addition, as the YjeF_N domain for the solution-state NMR experiments was expressed in D_2_O, not all backbone NH protons might have back-exchanged during the purification process, which could result in the absence of resonances in the solution-state ^1^H-^15^N NMR spectrum. Moreover, Ile-γ2 and Thr-γ methyl groups were not labeled for the solution-state NMR experiments and the corresponding resonances are thus not visible in the ^1^H-^13^C methyl TROSY spectrum. Finally, changes between the solution- and ssNMR spectra can result from differences in the measurement temperature (solid: 310 K, solution: 298 K) and sample preparation. Based on our experience on globular proteins, we however judge that these temperature effects only cause minor changes in the NMR spectra. In summary, based on a comparison of Yjef_N spectra of the Edc3 protein in solution and in the matured state, we conclude that the structure of the Yjef_N domain is largely maintained after LLPS.

### Interactions between the IDR and the Yjef_N domain are crucial for LLPS

Next, we assessed the importance of specific regions of Edc3 for LLPS and maturation. To that end, we performed in vitro experiments to determine to what degree various versions of Edc3 undergo LLPS and with what rate the condensed phase matures (Fig. 3b). In detail, we induced LLPS of Edc3-containing complexes and then measured over time the fraction of the total protein that is (i) not phase-separated, (ii) has undergone LLPS, and (iii) has matured into an insoluble gel (see methods). These data show that the Edc3 protein at 25 mm NaCl (Fig. 3b, panel 1) fractionates such that ~ 50% of the total protein is soluble (blue line at timepoint 0), ~ 40% is in a condensed phase separation state (green line) and ~ 10% is in a matured state (red line). Over the course of an hour, the LLPS state matures and transforms into the insoluble matured state, such that ~ 50% of the protein is then in a soluble state (blue) and ~ 50% of the total protein is in the matured phase (red). Note that the concentration of the soluble (blue) protein is constant at the critical concentration that is required for the formation of macroscopic phase-separated droplets, whereas the protein that is in the LLPS phase (green) at time 0 is fully transformed into the matured gel-like state (red). At higher salt concentrations (Fig. 3b, panels 2 and 3), the critical concentration for LLPS increases and less protein participates into a LLPS state. As a result, the fraction of the protein that ends up in the matured state is lower at higher salt concentrations. From these data, we conclude that electrostatic interactions play an important role in the LLPS process of the Edc3 proteins. Likewise, also upon addition of 5% 1,6-hexanediol, the phase separation propensity of Edc3 is reduced (panel 4), which indicates that hydrophobic interactions have a role in the Edc3 LLPS process. Interestingly, the addition of salt or 1,6-hexanediol not only increases the critical concentration for LLPS, it also appears to reduce the rate with which the LLPS protein matures. This indicates that the mode of the intermolecular interactions is similar in the LLPS and the matured state.

Based on the above setup, we addressed if the Edc3 LSm domain influences the LLPS and maturation process of the Edc3 protein. In agreement with the flexibility of the Edc3 LSm domain in the matured state of Edc3 (Fig. 1c–e), we observe that the removal of the Edc3 LSm domain has no significant effect of the LLPS extend and maturation rate (panel 5).

Previously, we have shown that the interaction between the Edc3 IDR and the Edc3 dimerization domain is important for the LLPS process of Edc3. In particular, we have identified two regions in the IDR that can interact with the Yjef_N domain^25^. These two regions comprise the amino acids K90–K111 (termed IDR1, Fig. 3a) and L159–L172 (termed IDR2, Fig. 3a), respectively. To assess the relative importance of these segments for the self-assembly process of Edc3, we performed LLPS and maturation assays with versions of the protein that lack either the first (IDR1, Fig. 3b, panel 6) or the second (IDR2, Fig. 3b, panel 7) disordered segment that interacts with the Yjef_N domain (Fig. 3b). We found that removal of the first or second segment in the IDR strongly reduces the LLPS propensity of Edc3, where the first segment has a slightly stronger influence on LLPS than the second one. These results underscore the degeneracy in the interaction networks that are responsible for cellular phase transitions and identify IDR1 and IDR2 as important interaction sites for LLPS.

To support this notion, we prepared [^13^C, ^14^N] and [^12^C, ^15^N] mixed-labeled Edc3-∆LSm samples in the matured state and used these to probe intermolecular contacts between Edc3 dimers. Indeed, we observed intermolecular contacts between HN and HC atom pairs in NHHC experiments^39^ verifying the presence of intermolecular contacts between two Edc3 dimers. These contacts most likely result from interactions of the IDR of one Edc3 dimer with the YjeF_N domain of another Edc3 dimer (Supplementary Fig. 4).

### Details of the IDR and Yjef_N domain interactions

**Fig. 4:**
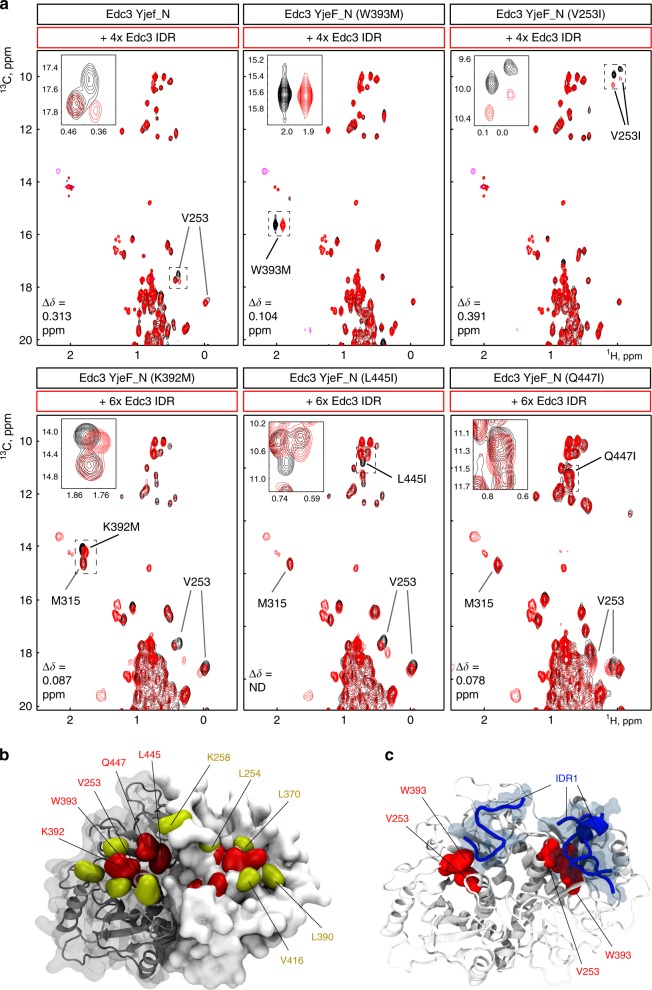
The IDR interacts directly with the YjeF_N domain. **a** The binding site of Edc3 IDR on the Edc3 Yjef_N domain was mapped using methyl TROSY NMR spectra. Specific residues on the Yjef_N surface were mutated into methyl group-containing amino acids to add NMR-active probes. These mutant proteins were subsequently used in NMR titration experiments. Chemical shift perturbations of the introduced methyl groups (Δ*δ* = √(Δ*δ*_C_^2^ + Δ*δ*_H_^2^) indicate if the corresponding residue is part of the Edc3 IDR binding site on the Edc3 Yjef_N domain and considered to be significant if Δ*δ* > 0.05 ppm (N.D.: owing to spectral overlap it was not possible to determine the extent of the CSP). Spectra for methyl groups that we found to be outside the IDR-binding site are shown in Supplementary Fig. 4. Note that the naturally occurring methyl groups of V253 are shifted owing to the W393M mutation and are thus not present at the WT resonance frequencies. In the V253I mutant protein, the methyl groups of V253 are not present and thus not detected in the spectra. In the W393M mutant, the V253 methyl groups are shifted to another spectra position and thus not readily identifiable. **b** The identified IDR-binding site is highlighted onto the Yjef_N homology model. Residues of the YjeF_N domain that interact with the IDR (W393, V253, K392, L445, or Q447) are colored red while non-interacting residues (L370, V416, L254, K258, and L390) are colored in yellow. **c** MD simulations on the complex formation between the YjeF_N dimer (in white) and peptides corresponding to the first YjeF_N interacting residue stretch of the IDR M93-E106 (IDR1). In the MD simulation, for three out of six replicas, the IDR peptides (in blue) directly interacted with residues V253 and W393 (in red) that were identified as YjeF_N-interacting hotspots in our solution-state NMR data. The figure shows the endpoints of the simulations after 100 ns

To address the interaction between the IDR and the Yjef_N domain with atomic detail, we turned to solution-state NMR techniques. In titration experiments, we observed clear chemical shift perturbations in ^1^H-^15^N TROSY and ^1^H-^13^C methyl TROSY NMR spectra of the Yjef_N dimerization domain when the IDR is added in *trans* (Supplementary Fig. 5a, b). To identify where the IDR interacts with the YjeF_N domain, we assigned a number of the resonances in the ^1^H-^15^N and ^1^H-^13^C spectra through a mutational approach (Fig. 4a). In particular, we were able to identify that W393 (based on ^1^H-^15^N data) and V253 (based on methyl TROSY data) in the dimerization domain are both part of the interface with the Edc3 IDR. As W393 and V253 are close in space on the surface of the Yjef_N domain they are part of the same binding pocket. To define additional residues that are part of this pocket we took advantage of a slightly modified version of the methionine scanning approach^40,41^. In this method, methyl group-containing residues were introduced in the spatial vicinity to W393 and V253. These methyl groups were subsequently used as probes for the interaction of the Yjef_N domain with the IDR. Based on this strategy, we were able to identify that K392, L445, and Q447 are also part of the IDR-binding pocket (Fig. 4a) as the introduced methyl groups experience chemical shift perturbations (CSPs) that are larger than 0.05 ppm. The introduced methyl groups did not alter the interaction mode between the Yjef_N domain and the IDR as the naturally occurring methyl groups of V253 underwent the same CSPs in the WT protein and the mutants. At the same time, we identified that K258, L254, L370, L390, and V416 are located outside the IDR-binding interface (Supplementary Fig. 5c), as these reporter methyl resonances are insensitive to the IDR, whereas the methyl groups of V253 remain effected by the IDR:Yjef_N interaction. In short, we find that the Edc3 IDR interacts with the dimerization domain at two specific pockets on the surface, one on each protomer (Fig. 4b).

To shed further light on how the IDR interacts with the YjeF_N domain, we used atomistic molecular dynamics (MD) simulations (Fig. 4c). To this end, we used a homology model of the YjeF_N domain (see Methods) and performed six simulations of the complex formation between the YjeF_N domain in dimeric state and a short peptide that corresponds to IDR segment M93-E106 (IDR1). For each MD simulation, two ‘IDR1 peptides’ were randomly placed in the aqueous phase above the YjeF_N dimer, shortly minimized and equilibrated with position restraints, and the system was eventually freely evolved for 100 ns. Interestingly, in three of the six runs, we observed that peptides (in blue in Fig. 4c) bound the YjeF_N domain in direct proximity of V253 and W393 (in red), corroborating the solution-state NMR data. Peptides that bound in proximity of these residues also remained stably associated with the YjeF_N domain (Supplementary Fig. 6, see also Supplementary Movie 1).

### RNA interacts with the IDR and thereby increases rigidity

**Fig. 5:**
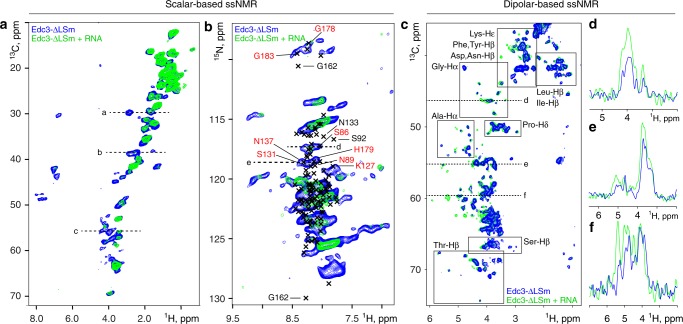
RNA interacts with the IDR and results in reduced dynamics in the matured state. **a**, **b** Scalar-based CH and NH spectra that detect molecular elements with fast nano-second motions recorded on Edc3-∆LSm in the absence (blue) and presence (green) of RNA, respectively. **b** The solution-state ^1^H-^15^N assignments of the IDR are indicated with black crosses. Putative interaction sites with RNA (red labels) or the YjeF_N domain (black labels) are indicated (see Fig. 3a). Dashed lines indicate 1D slices as represented in Supplementary Fig. 7. **c** Dipolar CH spectra that detect rigid, immobile molecular entities were recorded on Edc3-∆LSm in the absence (blue) and presence (green) of RNA. Several boxes indicate predicted chemical shifts for specific amino acids. **d**–**f** 1D slices of highlighted regions

Previously, we observed that RNA significantly enhances the LLPS tendency of the Edc3 protein as three distinct regions on the IDR can directly interact with RNA^25^ (Fig. 3a). Our data here show that RNA significantly enhances the rate with which the phase-separated Edc3:RNA complex matures (Fig. 3b; panel 8) as at the earliest timepoint, a large fraction of the protein already resides in the matured phase. Interestingly, for other proteins it has been shown that RNA prevents the formation of RNP granules^42^, which highlights that small changes in the system can result in different LLPS and maturation behavior. To obtain insights into the molecular mechanism by which RNA is embedded into the matured state of phase-separated Edc3, we conducted ssNMR experiments on Edc3–ΔLSm protein in the presence of RNA. Interestingly, based on scalar-based CH and NH spectra we observed that the incorporation of RNA into the protein-rich Edc3 phase results in a significant loss in ssNMR signals, consistent with a reduction of the internal dynamics in the matured phase (Fig. 5a, b, Supplementary Fig. 7; blue v/s green). In particular, we observe that many resonances that are typical for unstructured backbone- and side chain regions disappear upon interaction with RNA (Fig. 5b, green). This data thus directly reports on the interaction between RNA and the IDR, which results in a loss of mobility of the IDR (Fig. 5b). Using assignments obtained from solution-state NMR^25^ (black crosses), the regions where signals disappear upon RNA incorporation (red labels) include S86, K127, S131, N137, and G183. This confirms our previous solution-state NMR analysis on the putative IDR:RNA interaction site (Fig. 3a), as these residues were identified to directly interact with RNA. Furthermore, many resonances of the IDR that are known to interact with the Yjef_N domain are not observed in these ssNMR spectra, as these amino acids are rigid both in the absence and in presence of the RNA.

To independently confirm the rigidification of the IDR upon interaction with RNA, we recorded dipolar CH experiments on the matured state of the Edc3–ΔLSm protein in the absence and presence of RNA. In those experiments, we observe that a number of peaks appears upon addition of RNA, confirming the notion that residues of the IDR are stabilized by the RNA (Fig. 5c–f, green). The corresponding correlations (for example, at 5.39 ppm ^1^H and 59.9 ^13^C ppm) are consistent with lysine side chain and serine/threonine backbone ^1^H-^13^Cα peaks that are prominently present in the IDR segments that in solution interact with RNA (Supplementary Table 1). In conclusion, our ssNMR data suggest that RNA is recruited to the dynamic and unstructured IDR in Edc3, which results in decreased internal mobility.

## Discussion

LLPS is increasingly recognized as a cellular process that allows organizing cellular compartments across different spatial and temporal scales. A prominent example of such systems are RNP granules that contain proteins involved in translational repression and mRNA degradation^25^.

At present, the mechanisms by which LLPS modulates cellular function is not understood in sufficient detail.^1,43^ One potential consequence of LLPS is that the catalytic activity of the embedded enzymes is effected, potentially through changes in the local concentrations or accessibility of substrates^25^. To obtain knowledge of these aspects, it is essential to gain atomic-level insights into the structural organization of LLPS proteins. Here, we demonstrated the power of combining solid- and solution-state NMR to study the process of phase separation events on the structural and dynamical level and at atomic scale.

Our results on the central P-body protein Edc3 reveal that the different domains exhibit different levels of structural organization and dynamics after LLPS and a second transition into a matured phase (Fig. 1b). First, we find that the N-terminal LSm domain remains largely dynamic in the matured state, which leaves this domain accessible for contacts with additional P-body proteins including the Dcp1:Dcp2 mRNA decapping complex. Second, we find that the C-terminal YjeF_N domain largely retains its structure when the Edc3 protein undergoes a transition from the soluble to the matured phase-separated state. This is in strong contrast to other proteins that have been observed to form insoluble amyloidic fibril structures after phase separation^18^. The minor structural changes in the fold of the Edc3 protein reflects the fact that proteins that reside in cellular P-bodies can rapidly dissociate from these foci.

Within the phase-separated state, the Edc3 Yjef_N domain interacts with the IDR of Edc3. This transient interaction is likely intermolecular as it increases the network of interactions, leading to LLPS. Finally, we observe that the Edc3 IDR interacts with RNA in the matured state. These interactions involve distinct segments of the IDR and result in enhanced phase separation as the RNA extends and tightens the intermolecular interaction network.

**Fig. 6:**
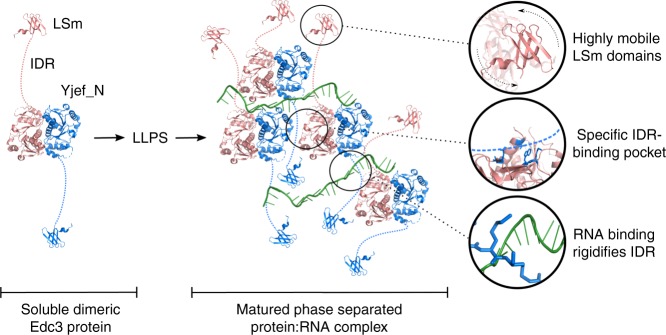
Model of Edc3- and RNA-mediated processing body formation. The soluble dimeric Edc3 protein (blue and red) contains an N-terminal LSm domain that is linked through an IDR with the C-terminal dimeric Yjef_N domain (left). Intermolecular interactions between RNA (green), the IDR and the Yjef_N domains (solution-state NMR) result in the establishment of an infinite interaction network that leads to liquid–liquid phase separation of the RNP. The LLPS proteins mature over time to form a gel-like state (Fig. 1b). Within this matured phase of processing bodies, the LSm domains remain highly flexible (solid-state NMR) and can thus interact with other processing body proteins (top circle). Based on solution-state and solid-state NMR data, we have shown that the Edc3 YjeF_N dimerization domain remains dimeric and interacts specifically via a surface patch with an Edc3 IDR of another Edc3 dimer (middle circle). The RNA interacts with the Edc3 IDR, which results in a rigidification of the unstructured region (bottom circle)

Based on our data, we can construct a model for the formation of the matured state of the Edc3 protein (Fig. 6). In this model, the dimeric soluble Edc3 protein engages in a number of intermolecular interactions that involve RNA. These contacts result in the formation of an indefinite interaction network that drives the transition of the Edc3 protein from the dimeric soluble form into a dense LLPS protein state that then further matures into an insoluble state. Interestingly, our data clearly show that the interactions between Edc3 and RNA that take place between the soluble components are, to a large degree, preserved in the matured phase-separated state. The structural differences upon LLPS are thus small, which facilitates the reversibility of the process and necessitates only small cellular perturbations to induce phase separations (Fig. 6).

Our work paves the way for future studies that address the exact structural changes that take place upon LLPS and those that result in the formation of the irreversible assemblies. Based on our data, these latter changes are small, but they will, nevertheless, have a significant effect on cellular homeostasis, as the maturation of cellular condensed protein phases needs to be prevented. We envision that our approach to study the structure and dynamics of LLPS complexes will be able to provide important insights in the current context and will also be applicable for other LLPS systems where irreversible assemblies are closely linked to fibril formation and disease.

## Methods

### Protein expression and purification

The genes for the full-length Edc3 protein, for Edc3 lacking the LSm domain (Edc3-∆LSm) and for the Edc3 YjeF-N domain from *Schizosaccharomyces pombe* were cloned into modified pET vectors that carried an N-terminal TEV-cleavable His_6_- or MBP-tag (Supplementary Table 2). The gene for the IDR of Edc3 was cloned into a modified pET vector that carried an N-terminal TEV-cleavable His_6_-GST-tag. Point mutations and deletions were introduced into the genes using standard site-directed mutagenesis methods (Supplementary Table 3).

*Escherichia coli* BL21 (DE3) Codon Plus RIL cells (Stratagene) were transformed with the appropriate plasmid and grown at 37 °C to an OD_595_ of 0.8 in LB. Overexpression was induced with 0.5 mm Isopropyl β-d-1-thiogalactopyranoside. After 18 h at 20 °C, cells were pelleted by centrifugation and resuspended in buffer 1 (25 mm sodium phosphate, pH 7.4, 250 mm NaCl, 1 mm DTT) complemented with 10 mm imidazole, 5 mm MgCl2, 0.1% Triton X-100, lysozyme and 0.2 U/mL DNase I. Cells were lysed with ultrasound and insoluble cell debris were removed from the lysate by centrifugation. The supernatant was bound to equilibrated Ni-NTA resin, which was washed subsequently with buffer 1 complemented with 10 mm imidazole. Bound protein was eluted from the resin with buffer 1 complemented with 300 mm imidazole. Tobacco etch virus (TEV) protease was added to the elution fractions to cleave the purification tag from the target proteins.

The Edc3 IDR was dialyzed overnight at 4 °C into buffer 2 (25 mm HEPES, pH 8.0, 50 mm NaCl, 1 mm DTT) supplemented with 0.5 mm ethylenediaminetetraacetic acid (EDTA). The dialysate was applied to a cation exchange column (HiTrap 5 mL SP FF, GE Healthcare) equilibrated with buffer 2. To separate the protein of interest from the cleaved GST-tag, a gradient from 0 to 35% buffer 3 (25 mm HEPES, pH 8.0, 2 m NaCl, 1 mm DTT) was applied over a volume of 100 mL. Fractions containing the target protein were pooled and the buffer was exchanged to buffer 4 (25 mm HEPES, pH 7.3, 125 mm NaCl, 1 mm DTT) using centrifugal filters.

MBP-tagged Edc3 protein was purified from the cleared cell lysate (buffer 2) by anion exchange chromatography (HiTrap 5 mL Q FF, GE Healthcare) using a gradient from 0 to 40% buffer 3 over a volume of 100 mL. Fractions containing the target protein were pooled and the buffer was exchanged to buffer 4 by dialysis for subsequent size exclusion chromatography.

Proteins carrying only an N-terminal His_6_-tag were directly dialyzed into buffer 4 supplemented with 0.5 mm EDTA. Purification to homogeneity was achieved by size exclusion chromatography on HiLoad 16/600 Superdex 75 (Edc3 IDR, Edc3 YjeF_N) or Superdex 200 (Edc3-FL and Edc3-∆LSm) columns (GE Healthcare) in buffer 4.

NMR-active proteins were obtained by overexpression in (50–100%) D_2_O-based M9 minimal medium supplemented with 0.5 g/L ^15^NH_4_Cl as the sole nitrogen source and either 2 g/L ^1^H^13^C-glucose for ssNMR samples or 4 g/L ^1^H^12^C-glucose for solution-state NMR samples. For labeling of the Ile-δ_1_, Met-ε, Val-γ_1_/γ_2_, and Leu-δ_1_/δ_2_ methyl groups within an U-[^2^H^12^C]-background, the medium was supplemented with α-ketobutyrate (3-^2^H_2_-4-^13^CH_3_; 60 mg/L), methionine (methyl-^13^CH_3_; 100 mg/L), α-ketoisovalerate (3-^2^H-3-(methyl-^13^CH_3_)-4-^13^CH_3_; 60 mg/L) 1 h prior to induction. For simultaneous labeling of Ala-β methyl groups, 2-^2^H-3-^13^C-l-alanine was added to the medium 20 min prior to induction.

### RNA in vitro transcription and purification

RNA in vitro transcription and purification was essentially carried out as described before^25^. In vitro transcription of a 30-mer RNA was accomplished using in-house purified T7 polymerase^44^ and two DNA primers as the DNA template (Supplementary Table 4). Transcribed RNA was purified under denaturing conditions using anion exchange chromatography on a DNAPac PA100 column (22 × 250 mm, Dionex) using a gradient from buffer 5 (20 mm Tris, pH 8, 5 m urea) to buffer 6 (as buffer 5, supplemented with 2 m NaCl). The purified RNA was precipitated from the pooled fractions using 0.7 columes of isopropanol, followed by desalting on a PD10 column (GE Healthcare) and SpeedVac concentration. The obtained pure dry RNA product was resuspended at concentrations required for subsequent experiments. The quality of the RNA was assessed using urea-polyacrylamide gel electrophoresis in 1 × TBE (89 mm Tris, pH 8.0, 89 mm boric acid, 2 mm EDTA). RNA was visualized by methylene blue staining.

### LLPS assays

Different versions of the Edc3 protein (FL, ΔLSm, ΔIDR1, or ΔIDR2) in the presence or absence of salt, 5% 1,6-hexanediol or RNA were prepared at a final concentration of 150 μm by dilution from a non-phase-separated stock solution that was at a high salt concentration. Subsequently, two 8 µL aliquots of the sample were taken. To aliquot one 2 µL 2.5 m NaCl was added to dissolve the LLPS protein completely. This aliquot was cleared from any insoluble (matured) protein by centrifugation, after which the protein concentration of the supernatant was determined. This reading provides a measure for the protein that is not matured. The second aliquot was cleared from LLPS proteins and from matured proteins by centrifugation, after which the concentration of the Edc3 protein was determined. This reading provides a measure for the protein that is not phase-separated or matured. The amount of the protein that resides in the matured phase was calculated based on the total protein concentration and the amount of the protein in the soluble + LLPS phases. These measurements were performed right after the preparation of the sample (0 min) and after 30, 60, 90, 120, and 180 min incubation at room temperature. For the Edc3:RNA mixture, the protein concentrations were determined via the Oregon green label that was attached to 20% of the total protein. All conditions contained 25 mm HEPES, pH 7.3, and 25 mm NaCl, unless indicated otherwise. The reported values are the average of three independent LLPS/maturations experiments (*n* = 3 for all timepoints and for all conditions). The indicated error is the standard deviation of the independent measurements.

### Solution-state NMR experiments

All solution-state NMR samples were in buffer 4 and contained 5% D_2_O. NMR spectra were recorded at 298 K on Bruker AVIII-500 and AVIII-800 spectrometers equipped with room temperature and cryo probe heads, respectively. NMR titration experiments were carried out with 0.05–0.1 mm ^15^N- and ILVM methyl-labeled protein (Edc3 YjeF) and a four- to sixfold excess of unlabeled protein (Edc3 IDR). NMR spectra were processed using the NMRPipe/NMRDraw software suite^45^. Figures displaying NMR spectra and protein structures were produced using NMRview (onemoonscientific.com) and Pymol (pymol.org), respectively. Solution-state NMR parameters are given in an overview in Supplementary Table 5.

### ssNMR experiments

Phase-separated Edc3 samples for ssNMR measurements were obtained by lowering the salt concentration of concentrated protein from 125 mm to 25 mm by dilution with buffer 5 (25 mm HEPES, pH 7.3, 1 mm dithiothreitol; DTT). For samples to contain RNA, the protein was supplemented with a 2.5-fold molar excess of 30-mer RNA and buffer 5. Protein and protein:RNA samples were incubated for 30 min at room temperature. Droplets were pelleted by centrifugation at 15,000 *g* for 15 min. The supernatant was discarded, and the gel was transferred to the ssNMR rotors.

ssNMR experiments were conducted using 3.2 mm and 1.3 mm triple-resonance (^1^H, ^13^C, and ^15^N) MAS probe heads in static magnetic fields of 9.4, 16.4, 18.8, and 22.2 T, corresponding to proton resonance frequencies of 400, 700, 800, and 950 MHz, respectively. Scalar-based correlation experiments utilized HC INEPT^35^ and 6 ms mixing C-C TOBSY^36^ transfer steps. Samples were chilled to approximately 278 K sample temperature and spun at a MAS rate of 9 kHz. ^1^H-detected experiments were performed at a MAS rate of 60 kHz and a sample temperature of ~ 310 K. Dipolar-based sequences were used with cross-polarization (CP) steps with an amplitude-ramp of 80–100% on ^1^H and 15 kHz PISSARRO^46^ decoupling during detection periods. For J-based experiments, decoupled HSQC^47^ sequences were used with a 4 and 3.35 ms INEPT transfer time for NH and CH transfers, respectively. NHHC experiments^39^ were performed on [^12^C,^15^N]/[^13^C,^14^N]-mixed-labeled samples in the matured state. Dipolar-based CP steps were used for magnetization transfer from ^13^C to ^1^H and from ^1^H to ^15^N. Proton-mixing times were from 0, 1, and 2 ms. ssNMR parameters are given in an overview in Supplementary Table 6.

### MD simulations

MD simulations to probe the interaction between the YjeF_N domain and the Edc3 IDR were run with the g54a7 force field^48^ and the GROMACS simulation package version 4.6.3.^49^ A 3D model of the dimeric YjeF_N domain was obtained using homology modeling^50^ based on the structure of the human protein (PDB:3D3K)^37^. A linear 14-meric peptide corresponding to the IDR stretch M93-E106 was built in Pymol (https://pymol.org/, version 1.3). For the starting state, peptides were placed within ~ 1 nm distance from the dimeric YjeF_N domain in a cubic aqueous box. The system was first energy minimized, afterwards equilibrated in an NVT ensemble for 100 ps with position restraints, then further equilibrated in a NPT ensemble for 100 ps with position restraints, and eventually freely evolved for 100 ns. In total, six replicates of 100 ns duration each with randomized initial peptide positions were run.

Contacts between IDR segment M93-E106 and the YjeF_N domain were quantified with the g-mindist tool of the GROMACS simulation package using a contact cutoff of 8 Å. Contacts were counted over the last 50 ns of the trajectory. As the YjeF_N domain is symmetric, the contacts for both monomers were added together. The final analysis is the summation of the intermolecular IDR–YjeF_N domain contacts from three replicas.

### Reporting Summary

Further information on research design is available in the Nature Research Reporting Summary linked to this Article.

## Supplementary information

Supplementary Information

Description of Additional Supplementary Files

Supplementary Movie 1

Reporting Summary

Source Data

## Data Availability

Data supporting the findings of this manuscript are available from the corresponding authors upon reasonable request. The Source Data underlying Fig. 3b, Fig. 4c, Supplementary Fig. 1b and Supplementary Fig. 6 are provided as a Source Data file.
