# Supplementary material for: Atomic-level insight into mRNA processing bodies by combining solid and solution-state NMR spectroscopy
Source: Nat Commun. 2019 Oct 4;10:4536. doi: 10.1038/s41467-019-12402-3 (PMC6778109; doi:10.1038/s41467-019-12402-3)
Supplement: Supplementary file 1 — Supplementary Information [file 41467_2019_12402_MOESM1_ESM.pdf]

Supplementary Information

Atomic-level insight into mRNA processing bodies by combining solid and solution-state NMR spectroscopy

Damman et al.

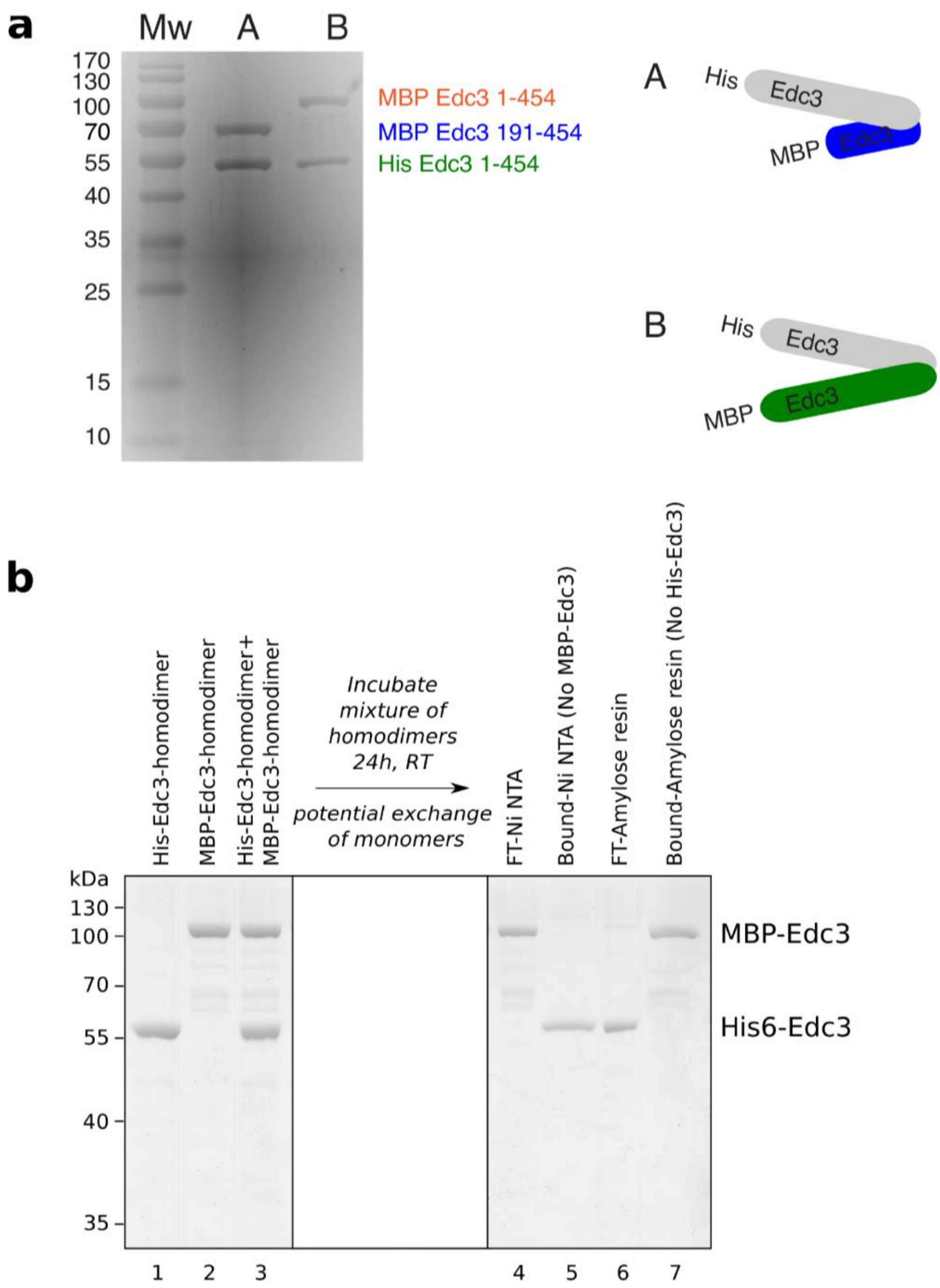

Supplementary Figure 1 | Edc3 forms a stable dimer in solution. (a) His-tagged full-length Edc3 was co-expressed in *E. coli* with either MBP-tagged Edc3 that contained Edc3 the Yjef_N domain (A) or with full length MBP-tagged full length Edc3 (B). The expressed proteins were purified in two steps. In step one all His-tagged Edc3 complexes (dimers) were selected for using Ni-NTA chromatography. In step two, all MBP-tagged Edc3 complexes (dimers) were selected for using amylose affinity chromatography. The Edc3 complexes that were purified this way thus contain at least one His-tag and at least on MBP-tag. The purified complexes were analyzed by SDS-PAGE and show a 1:1 ratio of a His-tagged and an MBP-tagged Edc3 monomer. This

clearly shows that the Edc3 protein forms a stable dimer in solution through the Yjef_N domain. Mw: molecular weight marker in kDa. **(b)** To assess the long-term stability of the Edc3 dimers, His-Edc3 homodimers (lane 1) and MBP-Edc3 homodimers (lane 2) were separately expressed and purified. These dimers were subsequently mixed in a 1:1 ratio (lane 3). After 24 hours at room temperature, one half of the mixed dimers was applied to a Ni-NTA column. The FT of this column contains all Edc3 dimers that do not contain a His-Edc3 monomer (lane 4). The elution of this Ni-NTA column contains all Edc3 dimers that contain at least one His-Edc3 monomer (lane 5). These latter dimers include MBP-Edc3 homodimers as well as (in case exchange of dimers took place) MBP-Edc3:His-Edc3 heterodimers. These mixed dimers are not present in the solution as the elution of the Ni-NTA column contains no MBP-Edc3 (lane 5). The second half of the incubated mixture of His-Edc3 and MBP-Edc3 homodimers was applied to an amylose column and a similar strategy was applied to probe for the occurrence of mixed dimers (lane 6,7). Taken together, we conclude that no exchange between Edc3 dimers takes place in 24 hours, indicating that the Edc3 dimers are very stable.

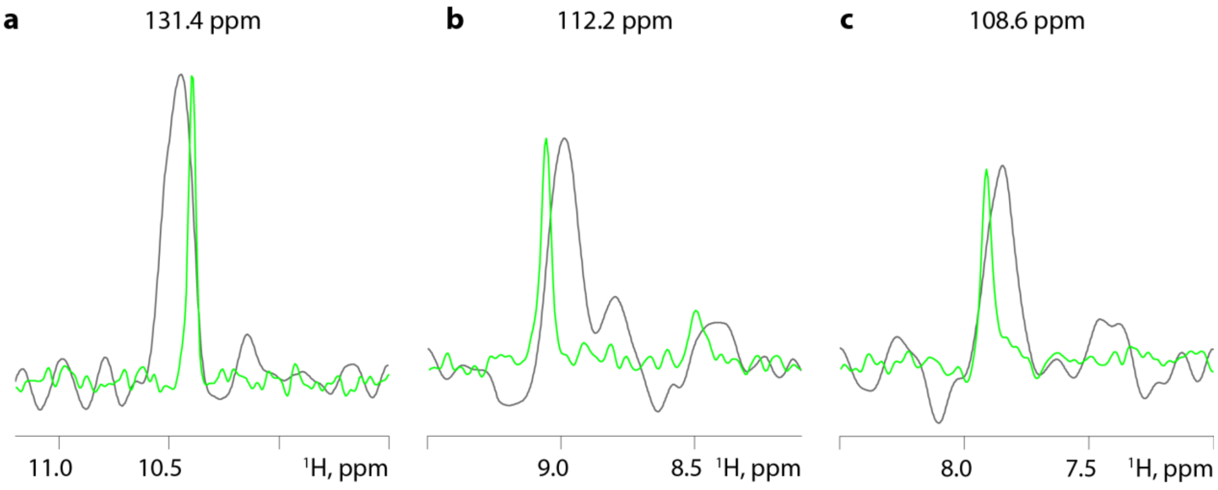

Supplementary Figure 2 | Comparison of 1D slices of NH spectra recorded by solution- or solid-state NMR. (a-c) 1D slices of resonances observed in both solution (green) and solid-state (gray) NMR spectra in Fig. 2a. Specific resonances are highlighted in Fig. 2a. Slices were scaled to match peak intensity.

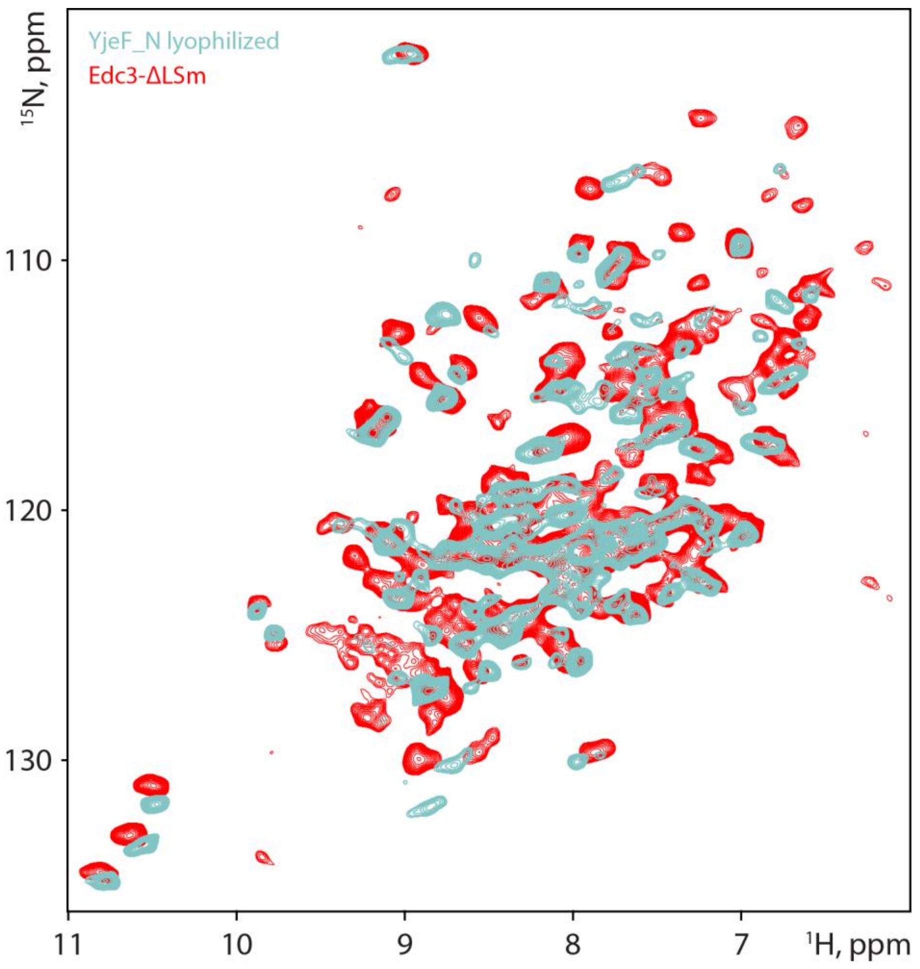

Supplementary Figure 3 | The fold of the YjeF_N domain in isolation is conserved in the matured state. Comparison of ^{1}H-detected dipolar NH (solid-state NMR) spectra recorded on Edc3-ΔLSm in the matured state (red) and lyophilized YjeF_N domain (cyan) highlighting the conservation of the fold of the YjeF_N domain. Differences between the two spectra can result from the IDR that interacts with the Yjef_N domain in the Edc3-ΔLSm construct as well as from differences in the ssNMR sample preparation.

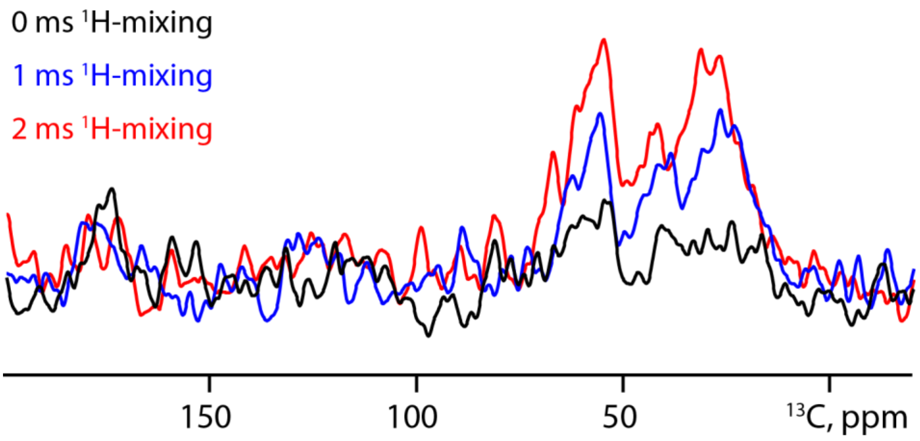

Supplementary Figure 4 | Edc3 dimers have intermolecular interactions during LLPS. NHHC spectra recorded on mixed ^{13}C-only labeled and ^{15}N-only labeled Edc3-ΔLSm with different proton mixing times shows the intermolecular interactions of Edc3-ΔLSm dimers in the matured state. Mixed ^{13}C-^{15}N Edc3 dimers are not present in the sample, as the dimers are highly stable (Supplementary Figure 1). The observed contacts are thus between protomers from two different Edc3 dimers. We have no indication that the Yjef_N dimers interact with each other, neither do we have an indication that two Edc3 IDRs interact with each other. Based on solution- state NMR data (Fig. 3a, 4a,b), we conclude that the Edc3 Yjef_N domain interacts with the Edc3 IDR. The inter-dimer contacts that we observe in the NHHC spectra are thus likely a result from interactions between the IDR of one Edc3 dimer with the Yjef_N domain of another Edc3 dimer.

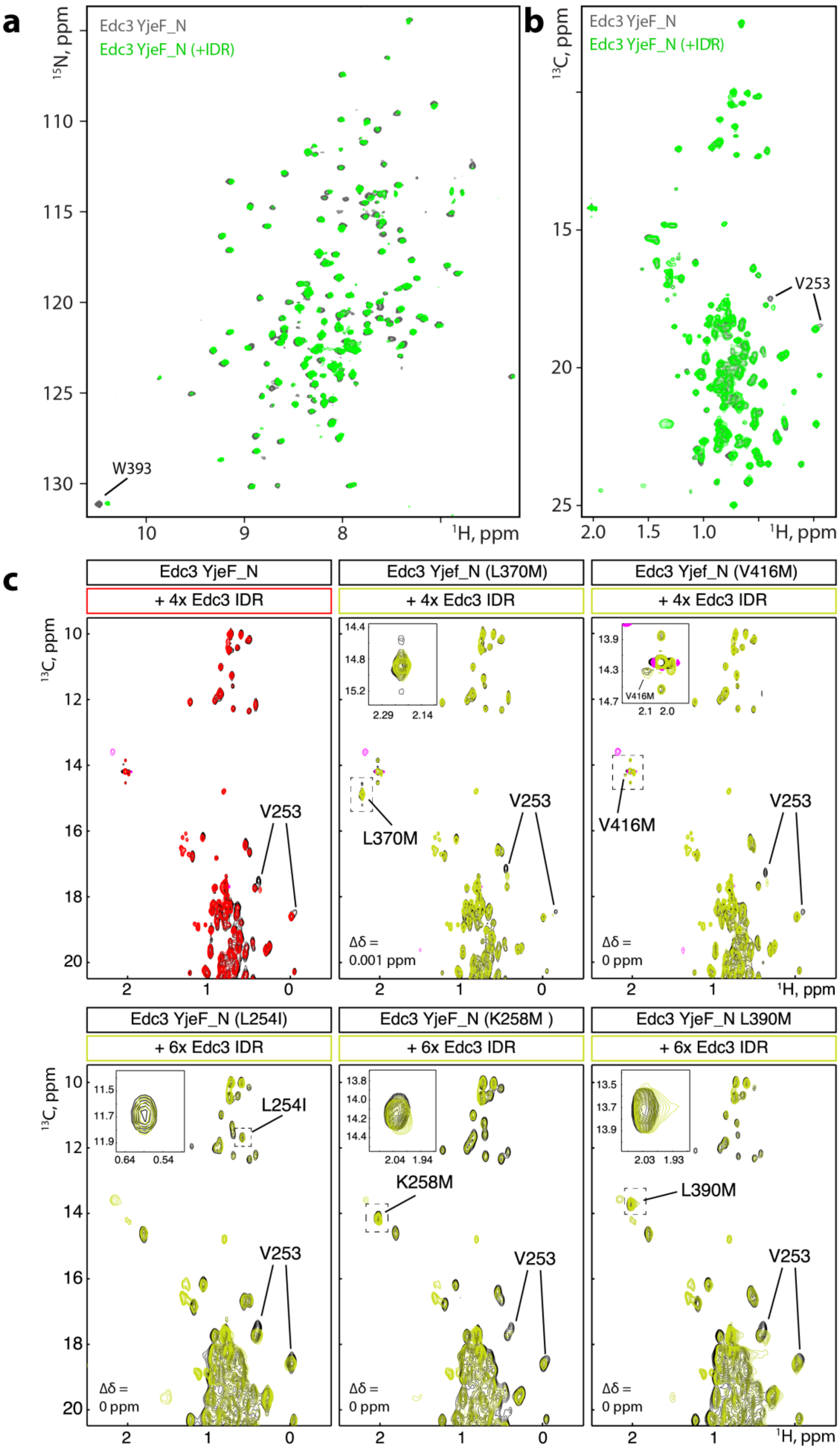

a
Edc3 YjeF_N
Edc3 YjeF_N (+IDR)
W393
^{15}N, ppm
^{1}H, ppm
b
Edc3 YjeF_N
Edc3 YjeF_N (+IDR)
V253
^{13}C, ppm
^{1}H, ppm
c
Edc3 YjeF_N
+ 4x Edc3 IDR
Edc3 Yjef_N (L370M)
+ 4x Edc3 IDR
Edc3 Yjef_N (V416M)
+ 4x Edc3 IDR
L370M
V416M
V253
Δδ = 0.001 ppm
Δδ = 0 ppm
Edc3 YjeF_N (L254I)
+ 6x Edc3 IDR
Edc3 YjeF_N (K258M)
+ 6x Edc3 IDR
Edc3 YjeF_N L390M
+ 6x Edc3 IDR
L254I
K258M
L390M

Supplementary Figure 5 | The Edc3 IDR interacts with the YjeF_N domain in solution. **(a)** Solution state ^{1}H-^{15}N TROSY and **(b)** ^{1}H-^{13}C HMQC spectra of the YjeF_N domain in solution in the absence (gray) or presence (green) of the unlabeled IDR. The assignment of W393 and V253 are highlighted as these residues show a significant chemical shift perturbation upon interaction with the IDR. **(c)** Introduced methyl groups on the Yjef_N surface that are not part of the IDR binding site (L370M, V416M, L254I, K258M, L390M). Note that the IDR interacts properly with the Yjef_N domain in all cases, as the naturally occurring methyl groups of Val 253 experience chemical shift perturbations comparable to the WT protein (panel b and Fig 4a).

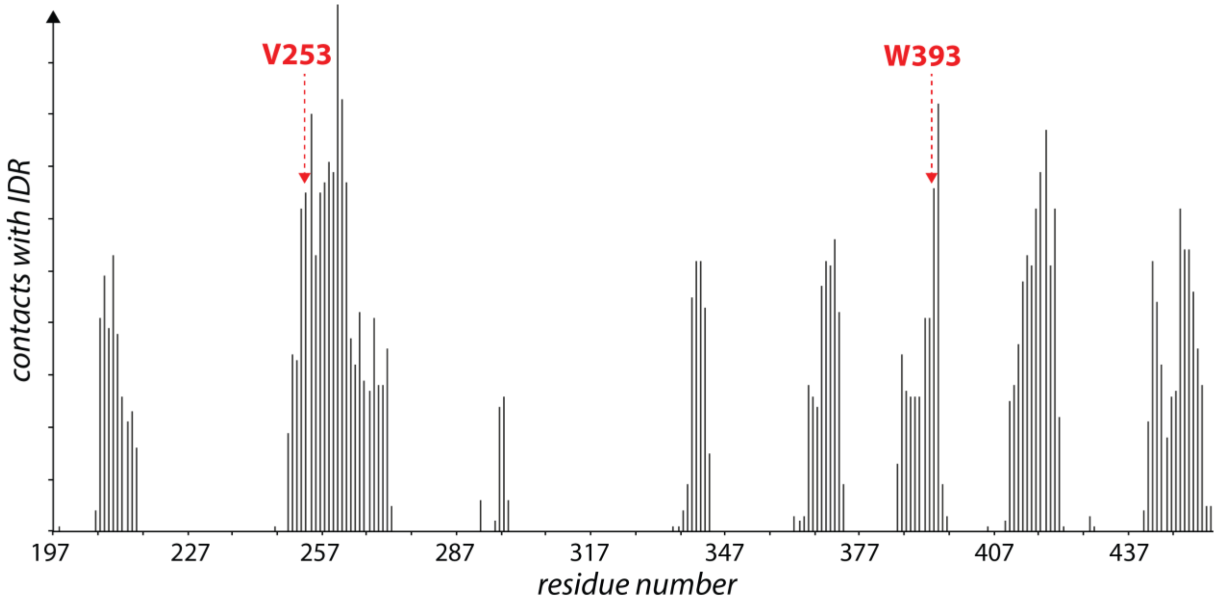

Supplementary Figure 6 | Analysis of molecular dynamics simulations. The analysis of molecular dynamics simulations results in the identification of the binding epitope that was also determined based on solution-state NMR experiments (in red in Fig. 4c). The contacts between the IDR and the Yjef_N domain were summed over the last 50 ns of the MD simulations. Details of the MD results are provided as a Source Data file.

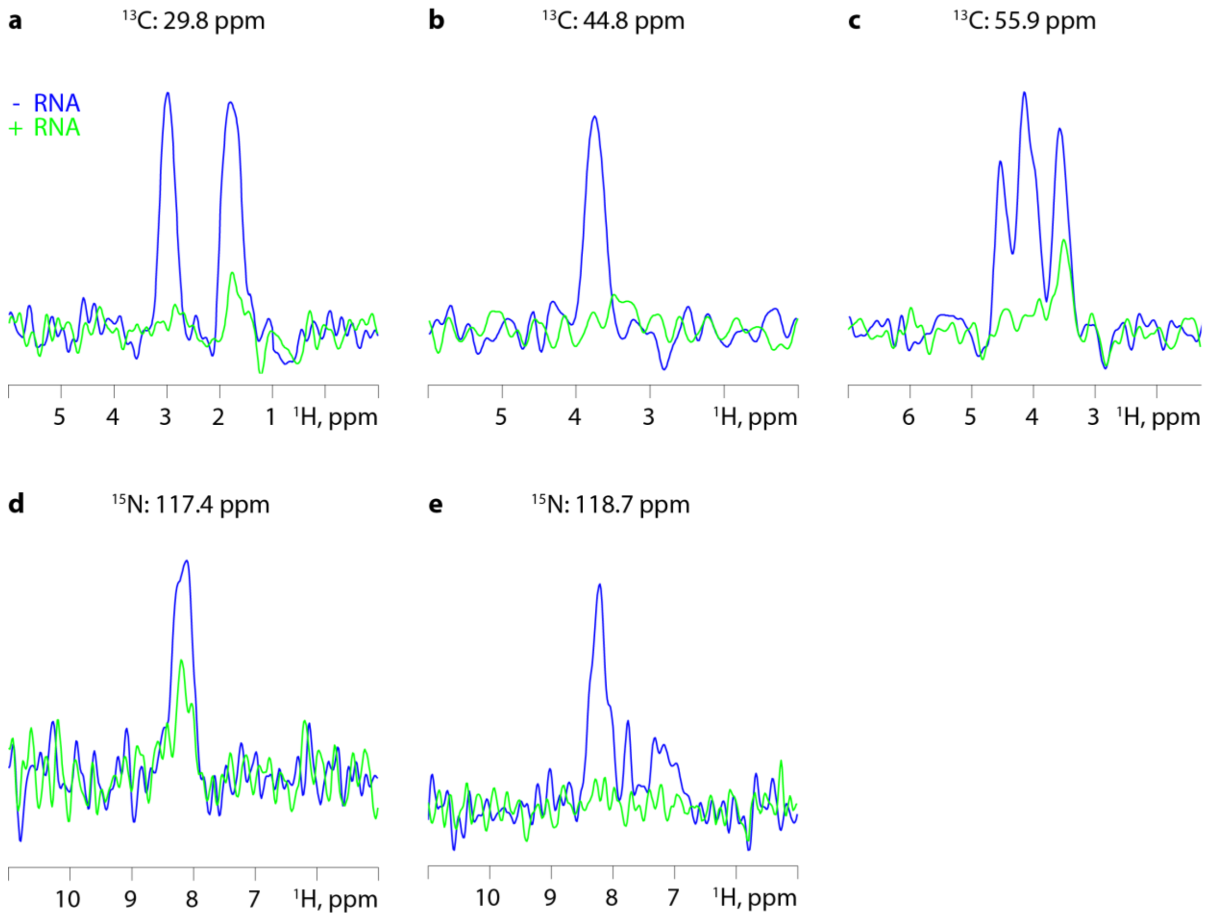

Supplementary Figure 7 | RNA stabilizes dynamic unstructured domains of Edc3. (a-c) 1D slices of resonances with reduced intensity after addition of RNA (green) compared to the absence of RNA as observed in the scalar-based CH spectra (Fig. 5a) and **(d,e)** scalar-based NH spectra.

Supplementary Table 1 | Amino acid distribution and occurrence for the Edc3 YjeF_N domain and IDR.

	YjeF	*IDR*
Ala	26	7
Arg	6	3
Asn	14	9
Asp	10	9
Cys	3	1
Gln	16	3
Glu	8	8
Gly	18	6
His	7	4
Ile	18	2
Leu	33	8
Lys	10	20
Met	1	1
Phe	9	6
Pro	11	6
Ser	19	18
Thr	21	5
Trp	3	1
Tyr	3	2
Val	23	7

Supplementary Table 2 | Primer sequences of Edc3 cloning.

Primer	Sequence	Purpose
Edc3_NcoI_1_f	CATGCCATGGGTATGTCTGTAGCTGATTTTTATG	Cloning of Edc3 FL
Edc3_NcoI_72_f	GGCCATGGGTCCGAGTACAAATTCTACCAAGTTG	Cloning of Edc3 IDR and ΔLSm
Edc3_BamHI_194_r	CGGGATCCTCATTTCTTTAATGTTTCACGGCTG	Cloning of Edc3 IDR
Edc3_NcoI_195_f	GGCCATGGGCAAGCCATTCGTCGATGAAAA	Cloning of Edc3 YjeF
Edc3_BamHI_454_r	CGGGATCCTCAGTTAGTGCTGGTACATG	Cloning of Edc3 FL, YjeF and ΔLSm
IDR_del90-110_f	CGGAAACTTATTCTTCAAAGAACAAAACAAGTTTTCGCTGAATTTCG	Cloning of Edc3 ΔIDR1 mutant
IDR_del90-110_r	CGAAATTCAGCGAAAACTTGTTTTGTTCTTTGAAGAATAAGTTTCCG	
IDR_del158-171_f	CTGTTAAAGACGAATTTGTAGATTTAAGTTCTTCAAGCAATGGAC	Cloning of Edc3 ΔIDR2 mutant
IDR_del158-171_r	GTCCATTGCTTGAAGAACTTAAATCTACAAATTCGTCTTTAACAG	
YjeF_M-2G_fwd	CTTTATTTTCAGGGCGCCGGCGGCAAGCCATTCGTC	Binding site mapping
YjeF_M-2G_rev	GACGAATGGCTTGCCGCCGGCGCCCTGAAAATAAAG	
YjeF_V253I_fwd	CTTTCTCAATTTGTTTTTTCAATCTTAGGAGGACATAAACG	
YjeF_V253I_rev	CGTTTATGTCCTCCTAAGATTGAAAAAACAAATTGAGAAAG	
YjeF_L254I_fwd	CTCAATTTGTTTTTTCAGTCATTGGAGGACATAAACGATTGTC	
YjeF_L254I_rev	GACAATCGTTTATGTCCTCCAATGACTGAAAAAACAAATTGAG	
YjeF_K258M_fwd	CAGTCTTAGGAGGACATATGCGATTGTCTTCGAGG	
YjeF_K258M_rev	CCTCGAAGACAATCGCATATGTCCTCCTAAGACTG	
YjeF_L370M_fwd	GGCTAATGATTTAAACGTTATGATACTTTCGGTAGACATC	
YjeF_L370M_rev	GATGTCTACCGAAAGTATCATAACGTTTAAATCATTAGCC	
YjeF_L390M_fwd	CGGTACAAAAAAAAAAACACGGCTATTATGCCCAAATGGACTTTGG	
YjeF_L390M_rev	CCAAAGTCCATTTGGGCATAATAGCCGTGTTTTTTTTTTGTACCG	
YjeF_K392M_fwd	CACGGCTATTTTGCCCATGTGGACTTTGGCTTTAGGG	
YjeF_K392M_rev	CCCTAAAGCCAAAGTCCACATGGGCAAAATAGCCGTG	
YjeF_W393M_fwd	GCTATTTTGCCCAAAATGACTTTGGCTTTAGG	
YjeF_W393M_rev	CCTAAAGCCAAAGTCATTTTGGGCAAAATAGC	
YjeF_V416M_fwd	GTTAAACAAGCTGCTGGTATGTCTGTATTTGTTGGAAAC	
YjeF_V416M_rev	GTTTCCAACAAATACAGACATACCAGCAGCTTGTTTAAC	
YjeF_L445I_fwd	CAAGTAACCGGCCAGTATATTGCCCAAATCTCATGTAC	
YjeF_L445I_rev	GTACATGAGATTTGGGCAATATACTGGCCGGTTACTTG	
YjeF_Q447I_fwd	GCCAGTATCTTGCCATCATCTCATGTACCAGCAC	
YjeF_Q447I_rev	GTGCTGGTACATGAGATGATGGCAAGATACTGGC	

Supplementary Table 3 | Protein constructs used in this study

Residue number / domain / mutations	Solubility / purification tag	Figure
1-454	N-His$_6$-TEV	1c, 1d, 1e, 3b, S1
72-194 (IDR)	N-His$_6$-GST-TEV	4a
195-454 (YjeF_N)	N-His$_6$-TEV	2a, 2b, 4a
YjeF_N (M-2G, L254I)	N-His$_6$-TEV	Supplementary Figure 5c
YjeF_N (M-2G, K258M)	N-His$_6$-TEV	Supplementary Figure 5c
YjeF_N (M-2G, L390M)	N-His$_6$-TEV	Supplementary Figure 5c
YjeF_N (M-2G, K392M)	N-His$_6$-TEV	4a
YjeF_N (M-2G, W393M)	N-His$_6$-TEV	4a
YjeF_N (M-2G, L445I)	N-His$_6$-TEV	4a
YjeF_N (M-2G, Q447I)	N-His$_6$-TEV	4a
YjeF_N (V253I)	N-His$_6$-TEV	4a
YjeF_N (L370M)	N-His$_6$-TEV	Supplementary Figure 5c
YjeF_N (V416M)	N-His$_6$-TEV	Supplementary Figure 5c
72-454 (IDR-YjeF_N)	N-His$_6$-TEV	1c, 1d, 2a, 2b, 5
72-454, Δ90-110 (IDRΔ1 – YjeF_N)	N-His$_6$-TEV	3b
72-454, Δ158-171 (IDRΔ2 – YjeF_N)	N-His$_6$-TEV	3b
1-454	N-MBP-TEV	Supplementary Figure 1
195-454 (Yjef_N)	N-MBP-TEV	Supplementary Figure 1

Supplementary Table 4 | RNA sequence used in this study

RNA	Properties	Sequence	Figures	Internal reference
30U15mer	GA-only, but single U at position 15	GGAGGAGAGGAAGGUAAGGGAAGAAAGAAG	5	#10 primer

Supplementary Table 5 | Overview of the solution-state NMR parameters

Experiment	Sample	Protein concentration	Scans	Acquisition Time	Window Function	Figure
NH TROSY	YjeF_N domain	100 µM	64	50 ms / 28 ms	QSINE (2)	Fig. 2a
Methyl TROSY	YjeF_N domain	100 µM	64	48.128 ms / 31.807 ms	QSINE (2)	Fig. 2b
Methyl TROSY	YjeF_N domain point mutants: V253I, L370M, V416M, W393M	100 µM	64	48.128 ms / 31.807 ms	QSINE (2)	Fig. 4 + Supplementary Figure 5
Methyl TROSY	YjeF_N domain point mutants: K259M, K392M, L254I, L390M, L445I, Q447I	50 µM	128	58.368 ms / 25.4453 ms	QSINE (2)	Fig. 4 + Supplementary Figure 5

Supplementary Table 6 | Overview of the solid-state NMR parameters

Experiment	Sample	Quantity	Scans	Acquisition Time (Direct-/indirect dimension)	Window Function	Figure
CC TOBSY	Edc3-FL	~ 5 mg	288	20 ms / 10 ms	QSINE (3)	Fig. 1c,d
CC TOBSY	Edc3-ΔLSm	~ 5.4 mg	288	20 ms / 10 ms	QSINE (2)	Fig. 1c,d
NH (Dipolar)	Edc3-ΔLSm Fraction Deuterated	~ 3.3 mg	480	30 ms / 15 ms	QSINE (6)	Fig. 2a
CH (Dipolar)	Edc3-ΔLSm Fraction Deuterated	~ 3.3 mg	352	19.7 ms / 7 ms	QSINE (3 and 6)	Fig. 2b and Fig. 5c-f
NH (Scalar)	Edc3-ΔLSm Fraction Deuterated	~ 3.3 mg	96	20 ms / 15 ms	QSINE (3)	Fig. 5b
CH (Scalar)	Edc3-ΔLSm Fraction Deuterated	~ 3.3 mg	96	20 ms / 6 ms	QSINE (3)	Fig. 5a
NH (Scalar)	Edc3-ΔLSm Fraction Deuterated + RNA	~ 3 mg	96	20 ms / 15 ms	QSINE (3)	Fig. 5b
CH (Scalar)	Edc3-ΔLSm Fraction Deuterated + RNA	~ 3 mg	96	20 ms / 6 ms	QSINE (3)	Fig. 5a
CH (Dipolar)	Edc3-ΔLSm Fraction Deuterated + RNA	~ 3 mg	480	21 ms / 6 ms	QSINE (4)	Fig. 5c-f
NH (Dipolar)	YjeF_N domain lyophilized	~ 4.8 mg	224	20 ms / 12.3 ms	QSINE (4.5)	Supplementary Figure 3
