# Supplementary material for: Atomic-level insight into mRNA processing bodies by combining solid and solution-state NMR spectroscopy
Source: Nat Commun. 2019 Oct 4;10:4536. doi: 10.1038/s41467-019-12402-3 (PMC6778109; doi:10.1038/s41467-019-12402-3)
Supplement: Supplementary file 2 — Description of Additional Supplementary Files [file 41467_2019_12402_MOESM2_ESM.pdf]

**Description of Additional Supplementary Files**

**File name:** Supplementary Movie 1

**Description:** Yjef_N – IDR interaction as seen by MD. Example of a 100 ns MD simulation of the dimeric YjeF_N domain and two IDR1 peptide segments (in red and yellow colours) in a periodic aqueous box. The IDR segments were initially placed about 1 nm distal from the domain. The red IDR1 segment rapidly binds in direct proximity of residues V253 and W393 (in blue and cyan, respectively) that were identified as IDR – YjeF_N interaction hot-spots by solution NMR experiments. Moreover, the movie shows that the YjeF_N domain and the interaction with the IDR1 segment are stable over the time-course of the trajectory.
